# Efficacy of Deep Brain Stimulation on the Improvement of the Bladder Functions in Traumatic Brain Injured Rats

**DOI:** 10.3390/brainsci10110850

**Published:** 2020-11-12

**Authors:** Chellappan Praveen Rajneesh, Jian-Chiun Liou, Tsung-Hsun Hsieh, Hung-Yen Chin, Chih-Wei Peng

**Affiliations:** 1 School of Biomedical Engineering, College of Biomedical Engineering, Taipei Medical University, Taipei 11031, Taiwan; pr2502@tmu.edu.tw (C.P.R.); jcliou@tmu.edu.tw (J.-C.L.); 2 Department of Physical Therapy and Graduate Institute of Rehabilitation Science, College of Medicine, Chang Gung University, Taoyuan 33302, Taiwan; hsiehth@mail.cgu.edu.tw; 3 Neuroscience Research Center, Chang Gung Memorial Hospital, Linkou 33305, Taiwan; 4 Department of Obstetrics and Gynecology, Taipei Medical University Hospital, Taipei 11031, Taiwan; chin671009@gmail.com; 5 Department of Obstetrics and Gynecology, School of Medicine, College of Medicine, Taipei Medical University, Taipei 11031, Taiwan; 6 Research Center of Biomedical Device, Taipei Medical University, Taipei 11031, Taiwan

**Keywords:** deep brain stimulation, PPTg, traumatic brain injury, weight drop, bladder dysfunction, urodynamic measurement, EUS-EMG

## Abstract

Objective: Traumatic brain injuries (TBIs) are a prime public health challenge with a high incidence of mortality, and also reflect severe economic impacts. One of their severe symptoms is bladder dysfunction. Conventional therapeutic methods are not effective in managing bladder dysfunction. Henceforth, a research endeavor was attempted to explore a new therapeutic approach for bladder dysfunction through deep brain stimulation (DBS) procedures in a TBI animal model. Methods: TBI in this animal model was induced by the weight-drop method. All rats with an induced TBI were housed for 4 weeks to allow severe bladder dysfunction to develop. Subsequently, an initial urodynamic measurement, the simultaneous recording of cystometric (CMG) and external urethral sphincter electromyography (EUS-EMG) activity was conducted to evaluate bladder function. Further, standard DBS procedures with varying electrical stimulation parameters were executed in the target area of the pedunculopontine tegmental nucleus (PPTg). Simultaneously, urodynamic measurements were re-established to compare the effects of DBS interventions on bladder functions. Results: From the variable combinations of electrical stimulation, DBS at 50 Hz and 2.0 V, significantly reverted the voiding efficiency from 39% to 69% in TBI rats. Furthermore, MRI studies revealed the precise localization of the DBS electrode in the target area. Conclusions: The results we obtained showed an insightful understanding of PPTg-DBS and its therapeutic applications in alleviating bladder dysfunction in rats with a TBI. Hence, the present study suggests that PPTg-DBS is an effective therapeutic strategy for treating bladder dysfunction.

## 1. Introduction

Traumatic brain injuries (TBIs) are a primary public health concern worldwide. They can be defined as non-degenerative, non-congenitally acquired insults to the brain from an external mechanical force, that most likely alter the normal functions of the brain and lead to transient or irreversible impairment of cognitive, physical, and psychosocial functions, with an additional diminished or altered state of consciousness [1]. Furthermore, prolonged treatment strategies and economic burdens in terms of health spending and loss of positive outcomes are astounding [2] Approximately 69 million individuals worldwide are estimated to sustain a TBI each year [3]. Road accidents are a noteworthy source of TBIs and bladder dysfunction is a major and significant outcome of it [4]. Customary current treatment strategies for bladder dysfunction in neurological patients, such as catheterization, antimuscarinic drugs, intradetrusor injections, and urinary diversion, have significantly contributed to a certain level of improvement in patients’ health and survival [5]. However, these conventional treatments have not recently been updated. In this regard, a new treatment strategy is needed to advance the field of neurourology, which must be capable of exploiting the full treatment potential.

Deep brain stimulation (DBS) is a promising, US Federal Drug Administration (FDA)-approved therapeutic strategy which involves delivering electrical impulses to targeted areas in the brain, through which it is capable of modulating neuronal excitability with beneficial results in clinical and animal models [6]. Hence, it serves as an alternative therapeutic measure for several neurological, neurodegenerative, and psychiatric disorders. The mode of action of DBS is still elusive; yet it is understood that it is capable of decreasing the firing action of neurons and affects axonal projections in the targeted area, which results in multifarious interactions between local and distant sites with specific characteristics that depend on anatomical and physiological aspects of the targeted area [7]. The pedunculopontine tegmental nucleus (PPTg) acts as a target region for DBS in Parkinson’s disease (PD) patients for regulating improper gait functions [8]. In addition, the PPTg also being as a target for several other neurological and neurodegenerative disorders [9] Hitherto, the neural connectivity between PPTg and bladder function has not been well explored; hence, we chose it as our target for the present study. As mentioned above, road accidents are a significant source of TBIs, and the weight drop model for inducing a TBI was chosen to mimic road traffic collisions or falls.

In our previous study, we successfully established a typical TBI rat model with chronic bladder dysfunction [1]. Based on our previous results, we further explored the delayed chronology of TBI-induced changes in bladder functions and alleviation of ailments with the aid of the cutting-edge technique of a targeted DBS strategy. Furthermore, urodynamic measurements can be used to evaluate recovery from the ailment and the efficacy of the DBS strategy in rats with a TBI.

## 2. Materials and Methods

### 2.1. Experimental Animals

In total, 28 male Sprague–Dawley rats obtained from BioLASCO Taiwan (Yilan, Taiwan) and weighing 250~280 g were used in the study. Rats were housed under a 12-h light–dark cycle with access to food and water ad libitum. Animal experiments were approved by The Institutional Animal Care and Use Committee (IACUC) of Taipei Medical University (IACUC approval no. LAC-2013-0199).

### 2.2. Experimental Design

**Figure 1:**
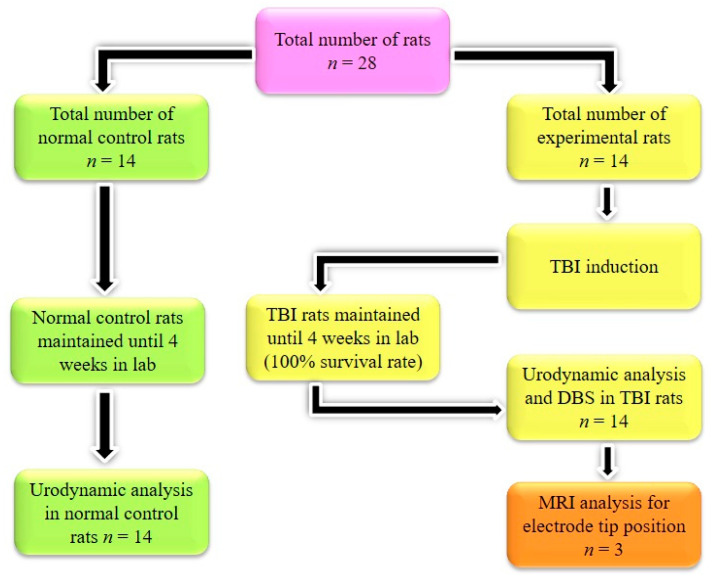
Schematic representation of the study design.

All animals were randomly assigned to two groups: a normal control (NC) group (*n* = 14) and an experimental group (*n* = 14). Experimental animals initially underwent an experimental TBI. Rats with a TBI that survived were housed for 4 weeks under proper care. At the end of the 4th week, rats with a TBI were subjected to stereotaxic surgery for the DBS procedure. Initially, rats with a TBI underwent a urodynamic analysis to check the delayed effects of the TBI on bladder functions. Later, standard DBS procedures were executed in TBI rats. Subsequently, the animals underwent a urodynamic analysis to evaluate the efficacy of DBS on bladder function (Figure 1). NC animals did not receive a TBI or participate in the DBS experiment. After the experiments, the rats were euthanized with an overdose of anesthetic drugs.

### 2.3. Induction of a TBI

Experimental animals were given 3% isoflurane (Aesica, Queenborough, Kent, UK) in 100% oxygen at 1 L/min for less than 10 min before the impact to minimize suffering. In preparation for the trauma, the animal was placed in a prone position over a sponge platform to expose the coronal sutures for the impact. Marmarou’s impact acceleration model [10,11] was used to induce the TBI with appropriate modifications. It consisted of a free-falling 450-g brass weight from 2 m in height through a vertical transparent Plexiglas tube. After the impact, ventilator support (Harvard Rodent Ventilator, model 683, South Natick, MA, USA) was employed with a gas mixture of 30% O_2_ and 70% N_2_O to avoid trauma-induced respiratory depression and death. Rats were maintained over an adjustable heating pad with the temperature set to 37.0 ± 0.5 °C, connected to a circulating water blanket until they had recovered from anesthesia. The rats were housed immediately at the end of the procedure. No mortality occurred in the present study.

### 2.4. The Brain Surgery Procedure for DBS

All rats were anesthetized with urethane (1.25 g/kg, subcutaneously; Sigma Aldrich, St Louis, MO, USA). A heating pad was used to maintain a constant body temperature as mentioned above, and a rectal probe was also used to monitor the body temperature of the animal until the end of the experiment. The surgical site was prepared for surgery by a required disinfection procedure. Later, the rat’s skull was exposed by cutting the skin over the middle of its head. Subsequently, the bregma point was carefully exposed with the aid of a stereotactic apparatus (Stoelting, Wood Dale, IL, USA).

**Figure 2:**
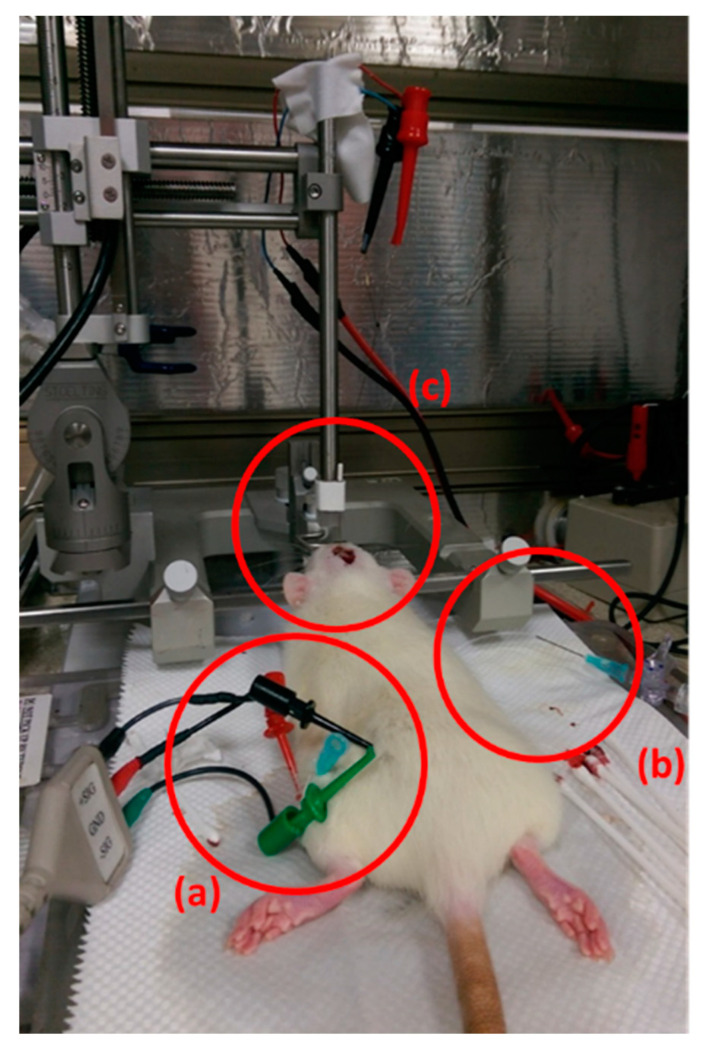
Surgical procedures performed in traumatic brain injury (TBI) deep-brain stimulation (DBS) rats (**a**) external urethral sphincter signal measuring electrodes, (**b**) bladder pressure measurement and micro tape fixed injection lines, (**c**) electrode after the positioning in the target area to deliver DBS.

The target area (PPTg) was localized from the bregma at a deviation of anterior–posterior (AP) −7.3 mm, mediolateral (L) +2.0 mm, dorsal–ventral (DV) −7.5 mm [12,13]. With the aid of an electric drill, a precise burr hole was made to embed the twisted bipolar electrode (SS80SNE-100, MicroProbes, Gaithersburg, MD, USA) into the targeted brain region (Figure 2). For the DBS procedure, the frequency was fixed at 50 Hz, and variations in the current-voltage of 1, 1.5, 2, and 2.5 V were applied along with a pulse width of 182 µs. The electrical stimulation was automatically applied at the onset of the reflexive bladder contraction, beginning when the bladder contraction pressure exceeded 25 cmH_2_O and lasting for 10 s to augment the bladder contraction. The DBS trials with amplitudes ranging from 1 to 2.5 V were conducted in a randomized amplitude sequence. The stimulation procedure described above was continually maintained until the end of the experiment for TBI rats. NC rats had an electrode Implanted but were not subjected to the stimulation procedure.

### 2.5. CMG and EUS-EMG Measurements

Cystometric measurements (CMG) were performed the same as in our previous studies [1,4,14]. The investigator who assessed the bladder activity was blinded to the status of the rat. The bladder of a rat was surgically exposed, and a polyethylene tube (PE60) (1.0 mm inside diameter and 1.5 mm outside diameter) was inserted in the bladder via the lumen for bladder pressure measurements and attached to both a pressure transducer and a syringe pump for filling cystometry (Figure 2). To measure external urethral sphincter electromyography (EUS-EMG), two 50-µm epoxy-coated platinum-iridium wire electrodes (A-M Systems, Everett, WA, USA) were pinned down at the tip of a 30-gauge needle and rooted into either side of the EUS under direct visual examination (Figure 2). The remaining free ends of the wire electrodes were connected to a computer for data analysis. Cystometric measurements with simultaneous DBS interventions were conducted in TBI animals after 1 h post-surgery to estimate the efficacy of DBS in TBI rats. The bladder was emptied after every cystometric trial with 10 min of equilibration before filling again. After the bladder was thoroughly emptied, cystometry was done at an infusion rate of 0.12 mL/min with a physiological saline infusion at room temperature.

Bladder activity was observed by CMG readings [1,4,14], and several CMG parameters were documented to further investigate the TBI’s effects on voiding activity, such as the voiding threshold (VT) (saline volume which is sufficient to induce bladder contractions); contraction amplitude (CA) (maximal intravesical pressure in the course of voiding); contraction duration (CD) (bladder contraction duration/period at the time of voiding); and intercontraction interval (ICI) (interval between micturition). The residual volume (RV) was measured by emptying the bladder achieved by applying manual pressure to the abdominal wall. The voided volume (VV) was calculated as the VT subtracted from the residual volume (RV) of saline introverted through the intravesical catheter after the final voiding contraction. The voiding efficiency (VE) was expressed by the formula VE = (VT − RV)/VT. Simultaneously, various EUS-EMG parameters were also documented, as previously described [1,4,14] including the burst period (BP), active period (AP), and silent period (SP). All of the CMG and EUS-EMG parameters were documented and calculated using Acknowledge software (BIOPAC Systems, Goleta, CA, USA).

### 2.6. Magnetic Resonance Imaging (MRI) to Assess the Electrode Position

To determine localization of the electrode position, three DBS rats were recruited from the TBI group after the aforementioned experimental trials. All anesthetized rats were placed in a prone position in a 7T Bruker PharmaScal 70/16 (Bruker Medical System, Karlsruhe, Germany). In this study, T2-weighted coronal MRI sequences (repetition time/echo time = 4500/80 ms) were utilized to confirm the location of the tip of the DBS electrode. Before the procedure, the electrode was withdrawn from the brain tissue to avoid image artifacts in the results. All MRI data were processed using Paravision 6.0 software (Bruker Medical System).

### 2.7. Statistical Analysis

All results are presented as the mean ± standard deviation (SD). To compare the differences of bladder activity between NC and TBI rats without DBS, the independent t-test was applied. The effects of DBS intensities for the CMG measures were evaluated by one-way repeated measures analysis of variance (ANOVA) with DBS intensities (0, 1, 1.5, 2, 2.5 V) as a within-subject factor, followed by post-hoc Bonferroni tests used to compare DBS intensities in CMG parameters. A one-way analysis of variance (ANOVA) was used for the comparisons among NC and TBI rats with different DBS intensities, followed by Tukey’s honest significant difference post-hoc test performed to make pairwise comparisons between the NC group and TBI group with DBS.”. A significance level of <0.05 was adopted for all analyses. Statistical analyses were executed using GraphPad Prism 6 software (GraphPad Software, San Diego, CA, USA).

## 3. Results

### 3.1. CMG Activity

Cystometric measurements were carried out by CMG and EUS-EMG. The observed CMG data of NC, TBI, and TBI-DBS rats are summarized in Table 1. During CMG, the average VT of the TBI and TBI-DBS rats was significantly increased compared to NC rats. The CA, CD, ICI, and VV of TBI rats exhibited significantly decreased values; however, TBI-DBS rats exhibited significant increases in these values compared to NC rats. The RV value of TBI rats increased by approximately 2-fold compared to the control; on contrary, TBI-DBS rats also exhibited an increased level of RV values against the NC rats, and the rats which received DBS of 50 Hz and 2.0 V exhibited a reduced level of RV compared to NC rats. The VE of TBI rats (39% ± 9%) was drastically reduced compared to NC rats (69% ± 6%); nevertheless, TBI-DBS rats which received stimulation of 50 Hz and 2.0 V exhibited a maximum elevated level of VE of 69% ± 8% (equivalent to that of control rats).

### 3.2. EUS-EMG Activity

**Figure 3:**
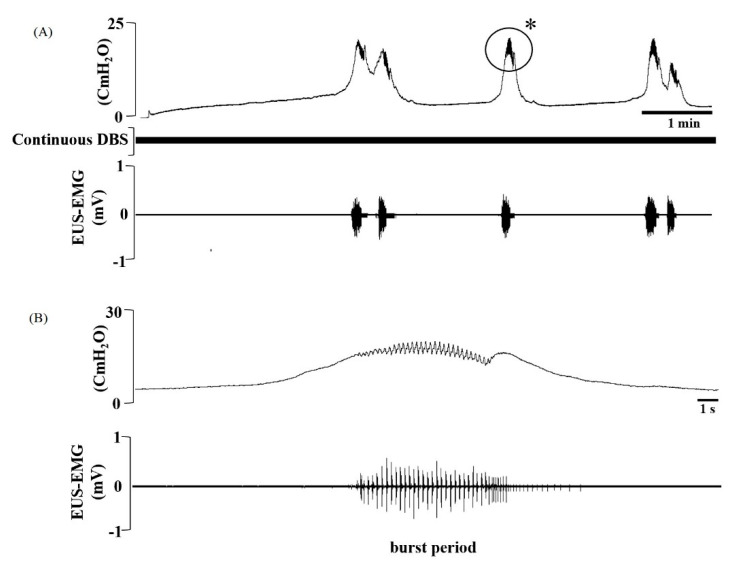
(**A**) The micturition cycle of 4 weeks of a traumatic brain injury deep brain stimulation (TBI-DBS) rat. Typical pattern of cystometric (CMG) (top) under continuous infusion and DBS (middle) with simultaneously recorded external urethral sphincter electromyography (EUS-EMG) (bottom) in a rat 4 weeks after a TBI. (**B**) Representative recording period of micturition at a faster timescale, and high-frequency oscillation (top) superimposed on the EUS-EMG (bottom). The burst period (BP) includes active period (AP) and silent period (SP). * magnified area.

Representative CMG and EUS-EMG tracings during continuous DBS from rats 4 weeks post-TBI (Figure 3). EUS-EMG measurements during voiding of NC, TBI, and TBI-DBS rats are displayed in Table 2. The BP decreased in TBI rats (3.41 ± 0.60 s) compared to that of NC rats (4.16 ± 0.48 s). However, TBI-DBS rats exhibited a significantly increased BP according to the variable electrical stimulation compared to TBI rats. The AP of TBI rats had significantly decreased (62.02 ± 2.94 s) compared to that of NC rats 93.05 ± 0.39 s (*p* < 0.05). Nevertheless, TBI-DBS rats exhibited a significant increase in AP according to the variable electrical stimulation compared to TBI rats. The SP of TBI rats displayed a significantly decreased value of 117.50 ± 4.28 s compared to that of NC rats (169.16 ± 3.97 s). On the other hand, the SP of TBI-DBS rats exhibited a significantly increased SP value according to the variable electrical stimulation compared to TBI rats. Yet, none of the BP, AP, or SP values of TBI-DBS rats was close to the corresponding EUS-EMG parameters in NC rats.

**Figure 4:**
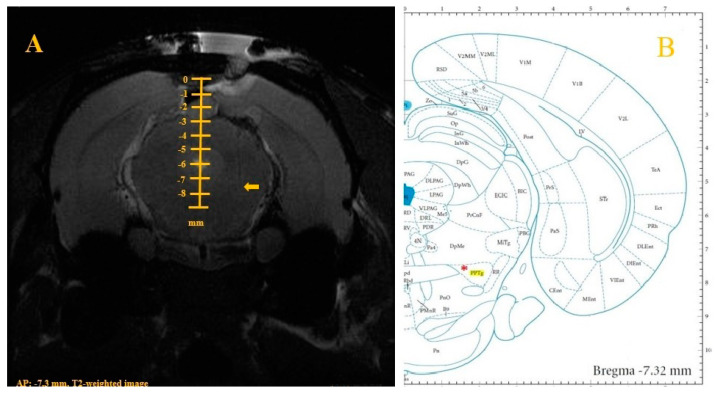
Example of a T2-weighted coronal image for measuring the target point of the tip of a deep brain stimulation (DBS) electrode in a rat with a traumatic brain injury (TBI). (**A**)—The tip of the DBS electrode was precisely located at the central point of the pedunculopontine tegmental nucleus (PPTg) (AP, −7.3 mm; L, +2.0 mm; and DV, −7.5 mm). The arrow indicates the electrode tip point. (**B**)—Schematic representation of the ventral PPTg in an atlas. The red asterisk indicates the ventral point of the PPTg (AP −7.3 mm, L +2.0 mm, DV −7.5 mm).

MRI results showed that the tip of the DBS electrode was precisely located in the ventral area of the PPTg (AP, −7.3 mm; L, +2.0 mm; DV, −7.5 mm) in TBI-DBS rats (100% accuracy rate), as shown in Figure 4. In consequence, our stereotaxic coordinate technique was reliable for appropriately localizing stimulation targets.

## 4. Discussion

The present study confirmed that TBI rats have altered micturition patterns. In TBI rats, VT values were remarkably increased, and CA values were strikingly reduced compared to NC rats. Bladder activity essentially depends on the sensitivity of afferent and central pathways involved in transduction, transmission, and integration of sensory information, which determines the threshold for reflex control and bladder activity [15,16]. It was observed that a TBI can induce neural dysregulation, although it might also affect afferent signal translation and transmission [17]. Since detrusor contractions entirely rely on sphincter relaxation, the massive hammer impact might have created extensive damage in the frontoparietal lobe of the brain, which might have affected signal conductivity. Hence, the increased VT values and decreased CA values might be an effect of improper synchronization between the sphincter and detrusor muscles due to the TBI.

On the other hand, with TBI-DBS, rats also exhibited increased values of VT compared to NC and TBI rats. However, CA values decreased after the differential intensity of electrical stimulation in TBI-DBS rats. The VT depends on the bladder capacity, and that is mainly governed by peripheral neurogenic afferent activity under CNS control. In the case of anterior cerebral lesions, tonic inhibitory control might be removed over the pontine micturition center (PMC), which ultimately results in a decreased bladder capacity and detrusor overactivity [18]. If the bladder has not sufficiently filled on account of insufficient afferent activation in the CNS, efferent nerve fibers might give rise to an impaired detrusor contractility state. Herein, even though a different voltage stimulation was used in this study, the applied voltage stimulation might not have been sufficient to activate the above-mentioned mechanism, and this might have resulted in increased levels of VT in TBI-DBS rats. Rats that received stimulation of 2.0 V exhibited comparatively decreased levels of VT, which shows that this particular voltage might be capable of activating afferent activity. The decrease in CA values might have been due to a response to differential electrical stimulation, which might act upon the suprapontine brain regions, and develop synchronization between the brain and detrusor muscle. Moreover, the DBS might have significantly improved the detrusor contraction force [4], which can be spotted by the increasing values of CA in response to higher stimulation intensities of 1.0~2.5 V.

CD levels decreased in TBI rats and increased in TBI-DBS rats. The decrease in the CA and CD levels indicates an underactive bladder with severe urinary retention conditions, i.e., unsuccessful bladder emptying or a urinary retention condition in TBI rats. Since typical micturition relies on the activation of the upper precentral gyrus, middle, upper frontal gyrus, thalamus, and caudal part of the anterior cingulate gyrus in the left hemisphere [19], TBI causes severe damage over the entire hemisphere and this could essentially control the efficacious bladder emptying, and impairment in this hemisphere eventually results in a deviating CD value. Besides this, the neuronal shock due to severe TBI would result in the transient diminishment of all reflexes. In the present study, the applied DBS voltage might have been sufficient to increase CD values in TBI-DBS rats by reestablishing the defective neuronal circuits and effectively increase the contraction force of the bladder walls.

VV and CD are highly interrelated [20]. The CD is highly dependent on the expelling efficiency of bladder contractions. Reduced CD levels in the present study indicate the poor expelling condition of the bladder. Hence, VV levels also decreased in TBI rats. In addition, the micturition process mainly relies on the cortical region of the brain. The TBI might have induced neural dysregulation over the cortical region of the brain that led to poor impulse conductivity in the neural network and ultimately to poor voiding conditions. Subsequently, the VV of TBI-DBS rats showed an increasing pattern under differential voltage stimulation conditions. Studies by Kesseler and colleagues [21] revealed that thalamic DBS can effectively change bladder functions, and might have eventually increased the VV in TBI-DBS rats.

ICI values we obtained were reduced, and RV values were increased in TBI rats. Both ICI and RV values are entirely based on effective bladder evacuation. After the trauma, the urinary excretion efficiency decreased; this was reflected in increased ICI values and reduced RV values in TBI rats. The TBI might have produced mixed damage mainly in the frontoparietal lobe and adjacent regions of the brain; hence, the lack of central inhibition of the PMC resulted in uninhibited bladder contractions [7]. In normal conditions, projections of the PMC act upon parasympathetic motor neurons that ultimately induce bladder contractions [22]. In the case of TBI rats, as we mentioned earlier, the PMC might have been heavily damaged; hence, it was evident that improper bladder emptying is a common phenomenon, and this might have been one of the reasons for decreased levels of ICI and RV in TBI rats. After DBS, ICI values significantly increased; subsequently, RV values decreased in TBI-DBS rats when compared with the TBI rats. At certain points, 50 Hz and 2.0 V of ES produce a much-reduced level of RV when compared to the NC rats. This might have been due to the effect of DBS, which might have improved neural regulation of bladder function leading to normalization from an abnormal bladder condition by influencing the PMC either directly or via descending connections [23]. Moreover, the increased ICI values and decreased RV values after DBS clearly show the effective bladder evacuation condition in TBI-DBS rats, which commendably was also reflected in the VE values of TBI-DBS rats.

VE values of TBI rats were strikingly reduced, whereas they were significantly increased in response to the different intensities of electrical stimulation in TBI-DBS rats. The decreased levels of VE observed in TBI rats were due to urinary retention; in other words, the bladder might not have completely emptied. As mentioned earlier, it is evident from acute changes in the post-TBI state that the bladder might have irregularities in regulating signal transmission and/or translation [7]. Therefore, complete bladder emptying could not be achieved. Hence, reduced VE levels were documented in TBI rats. As we stated above, stimulation levels used in the study might have been insufficient to evacuate urine from the bladder of TBI-DBS rats to achieve an increased VE, particularly when stimulation at 50 Hz and 2.0 V produced impeccable results of a 69% VE, which was equivalent to that of NC rats. A similar parameter in TBI rats with PnO-DBS also exhibited a faultless result [4] which represents the effectiveness of the intensities used in both the studies. To verify this, we observed values of ICI, RV, and VV in TBI-DBS rats, and the differential intensity of electrical stimulation significantly increased ICI and VV values, whereas it significantly reduced the RV values. All these parameters are interlinked with effective bladder evacuation, which ultimately increased the VE in TBI-DBS rats.

The tonic activity of the EUS depicts a shutting down of the ureteral outlet in the course of the urine-storage process, and burst activity produces a rhythmic opening and closing of the outlet to give pulsatile urine flow [24]. Results of the current study showed that the TBI differentially changed the BP, AP, and SP of EUS-EMG patterns. The EUS tonic activity of TBI rats decreased during bladder contractions, and a sudden surge was observed in the BP. Herein, the BP was comprised of the AP and SP; TBI rats invariably exhibited a typical phenomenon of a reduced bladder contractile condition and decreased urethral activity. Analogous to these observations, bladder contractility and reduced urethral activities were observed in TBI rats [25]. Remarkably, after DBS treatment with different intensities of electrical stimulation, BP values of TBI-DBS rats were elevated. From this observation, it was concluded that this remarkable improvement might have been achieved by DBS in the PPTg region.

EUS-EMG bursts and the SP duration, which respectively denote relaxation and opening of the outlet, are indispensable for attaining effectual voiding [26]. In the present study, the SP values of TBI rats were drastically reduced. Meanwhile, TBI-DBS rats exhibited a pattern of increasing SP values according to the different intensities of electrical stimulation. The decrease in SP values indicated that the urethra was open for a shorter period during voiding, which could contribute to the decreased levels of VE in TBI rats. The TBI we experimentally induced might have decreased SP values by distorting certain functions in the cortical region of the brain, which is responsible for the micturition process [25]. In addition, a gradual increase in SP was noted in TBI-DBS rats with different voltage stimulations, and this condition might have been due to neuromodulatory events, which could improve and reestablish neural connectivity in the targeted region.

Our results confirm that the weight-drop model produced reliable and stable TBI-induced bladder dysfunction in rats, and the DBS potentially acted upon the target region to improve the VE in rats with severe bladder dysfunction. In addition, quite a few limitations were unavoidable in this study. The present work is a voltage-dependent study associated with a neuromodulatory approach. Hence, we did not investigate cellular or biochemical aspects. Second, the effects of anesthetic drugs certainly influence urodynamic outcomes in rats. Herein, the results we obtained were significant in describing ailments and improvements after treatment. Finally, according to the results obtained from our previous study [12] on PPTg-DBS in normal rats (1.5 to 2.5 V) with almost similar intensities employed in the current study have indicated potentially inhibited micturition reflex contractions, thus the DBS procedure was evaded in NC rats. Therefore, further studies are warranted to overcome the current limitations of the present study.

## 5. Conclusions

This study disclosed a profound understanding of DBS and its therapeutic applications in a process to alleviate bladder dysfunction in TBI rats. DBS (at different voltages) and responses of bladder controls fostered a better understanding of neural regulation between the brain and urinary bladder. It was concluded from the findings that DBS was capable of inducing potential neural regulation that could control bladder functions in TBI rats. Consequently, the PPTg might be a promising target for the development of new therapies for TBI-related lower urinary tract dysfunction.

## Figures and Tables

**Table 1:** Cystometric measurements of normal control (NC), traumatic brain injury (TBI), and TBI rats treated with pedunculopontine tegmental nucleus (PPTg)-deep brain stimulation (DBS) at differential voltages of electrical stimulation (ES).

	VT (mL)	CA (cmH_2_O)	CD (s)	ICI (s)	RV (mL)	VV (mL)	VE (%)
**NC without ES**	0.38 ± 0.02	33.14 ± 2.64	23.78 ± 2.15	129 ± 3	0.16 ± 0.03	0.26 ± 0.02	69 ± 6
**TBI without ES**	0.53 ± 0.04 *	26.72 ± 3.13 *	17.63 ± 2.54 *	109 ± 4 *	0.32 ± 0.06 *	0.21 ± 0.04 *	39 ± 9 *
**TBI with 1.0 V**	0.56 ± 0.03 *	31.30 ± 3.45 *^ψ^	20.02 ± 3.28 *^ψ^	145 ± 7 *^ψ^	0.25 ± 0.02 *^ψ^	0.32 ± 0.03 *^ψ^	57 ± 6 *^ψ^
**TBI with 1.5 V**	0.55 ± 0.04 *	30.24 ± 3.02 *^ψ^	21.87 ± 3.41 *^ψ^	165 ± 7 *^ψ^	0.23 ± 0.02 *^ψ^	0.33 ± 0.04 *^ψ^	60 ± 7 *^ψ^
**TBI with 2.0 V**	0.49 ± 0.03 *^ψ^	31.18 ± 3.63 *^ψ^	23.05 ± 3.23 ^ψ^	171 ± 5 *^ψ^	0.14 ± 0.03 *^ψ^	0.34 ± 0.02 *^ψ^	69 ± 8 ^ψ^
**TBI with 2.5 V**	0.66 ± 0.06 *^ψ^	31.77 ± 2.40 *^ψ^	22.82 ± 2.71 ^ψ^	183 ± 6 *^ψ^	0.28 ± 0.03 *^ψ^	0.37 ± 0.05 *^ψ^	56 ± 1 *^ψ^

* Indicates a significant difference compared to the NC group; ^ψ^ Indicates a significant difference compared to TBI rats (week 4). Significance levels are marked at *p* < 0.05. Values are the mean ± standard deviation; *n* = 14 NC rats; *n* = 14 TBI rats without and with DBS at 50 Hz and 1.0~2.5 V. VT, voiding threshold; CA, contraction amplitude; CD, contraction duration; ICI, intercontraction interval; RV, residual volume; VV, voided volume; VE, voiding efficiency.

**Table 2:** External urethral sphincter electromyographic (EUS-EMG) activity in normal control (NC), traumatic brain injury (TBI), and TBI-deep brain stimulation (DBS) rats treated with pedunculopontine tegmental nucleus deep brain stimulation (PPTg-DBS) at different voltages.

	BP (s)	AP (ms)	SP (ms)
**NC**	4.16 ± 0.48	93.05 ± 0.39	169.16 ± 3.97
**TBI Week 4**	3.41 ± 0.60 *	62.02 ± 2.94 *	117.50 ± 4.28 *
**50 Hz, 1.0 V**	3.48 ± 0.66 *	63.28 ± 2.59 *	121.18 ± 3.71 *
**50 Hz, 1.5 V**	3.53 ± 0.68 *	68.61 ± 3.65 *^ψ^	135.71 ± 3.96 *^ψ^
**50 Hz, 2.0 V**	3.80 ± 0.31 *^ψ^	87.69 ± 2.88 *^ψ^	142.12 ± 2.98 *^ψ^
**50 Hz, 2.5 V**	3.64 ± 0.95 *	82.87 ± 2.66 *^ψ^	138.86 ± 3.18 *^ψ^

BP, burst period; AP, active period; SP, silent period. * Indicates a significant difference compared to the NC group; ^ψ^ Indicates a significant difference compared to TBI rats (week 4). The frequency of burst discharges is represented as a ratio between the number of silent periods and its burst period. Significance levels are marked at *p* < 0.05. Values are the mean ± standard deviation; *n* = 14 NC; *n* = 14 TBI rats without and with DBS at 50 Hz and 1.0~2.5 V.3.3. MRI assessment of DBS electrode localization.

## Abbreviations

Abbreviations	Expansions
NC	Normal control
TBI	Traumatic brain injury
DBS	Deep brain stimulation
EUS -EMG	External urethral sphincter electromyography
CMG	Cystometry
PPTg	Pedunculopontine tegmental nucleus
MRI	Magnetic resonance imaging
ANOVA	One-way analysis of variance
VT	Voiding threshold
CA	Contraction amplitude
CD	Contraction duration
ICI	Intercontraction interval
RV	Residual volume
VV	Voided volume
VE	Voiding efficiency
AP, L, DV	Anterior-posterior, mediolateral and dorsal-ventral
PMC	Pontine micturition center
CNS	Central nervous system
